# Clinical management of deep osseous defects in cases of peri-implantitis among indian patients

**DOI:** 10.6026/97320630018807

**Published:** 2022-09-30

**Authors:** Mohammed Ahsan Razi, Ankit Mahajan, Parveen Ranga, Puja Kumari, Jyoti Kumari, Ritika Saluja

**Affiliations:** 1 Hazaribag College of Dental Sciences & Hospital, Hazaribag, Jharkhand, India; 2 Government Dental College, Shimla, Himachal Pradesh, India; 3 Shaheed Hasan Khan Mewati Government Medical college Nuh, Haryana, India

**Keywords:** Peri-implantitis, dental implants, degranulation

## Abstract

Peri-implantitis is recognized as a complex pathology which could be defined as infectious inflammatory lesions that usually develop in the tissues around the implants. There are many protocols for the effective management of peri-implantitis that include mechanical debridement, the use of antiseptics and local/systemic antibiotics, and access and regenerative surgery formulated for the treatment of peri-implantitis. This study aims to evaluate the clinical outcomes of a mixed protocol for the regeneration of deep osseous defects. Records obtained from 27 patients who had already received treatment for peri-implantitis on one or more implants were retrospectively examined within the proposed time period between 24 and 30 months after their surgical treatment. A total of 33 implant sites were included and examined retrospectively. Descriptive statistics were calculated that include mean, SD, medians and confidence intervals at 95%. At the baseline, the mean Probing Depth was 8.19 ± 1.23 mm; Bleeding on Probing (BOP) was present on 29 out of 33 treated areas; pus was instead present on 17 out of 33 sites. At the time of final examinations, BOP was present on 9 out of 33 sites; pus was present only on two surgical sites. To conclude, a combined chemical-mechanical and regenerative decontamination therapy is effective in the treatment of peri-implantitis. Further investigation, which includes a control group and/or histologic findings, might be needed to ascertain the clinical results reported in the clinical studies.

## Background:

The currently emerging problem across the world is known to be peri-implant diseases which could be defined as infectious inflammatory lesions that usually develop in the tissues around the implants. [1-2] Peri-implant diseases can be effectively classified as peri-implant mucositis and peri-implantitis. [3] The term peri-implant mucositis is defined as a reversible inflammatory reaction of the soft tissues that surround the implant in function. In contrast, peri-implantitis is an inflammatory reaction associated with the loss of bone support around the implant in function. [4] The primary aetiological factor that is responsible for the development of peri-implant diseases is the accumulation of bacterial biofilms. [5] The unique key factor recognized for the diagnosis of peri-implant mucositis is the presence of bleeding on probing. [6-7] For the effective diagnosis of peri-implantitis, it is mandatory to combine it with an increase in probing depth related with bone loss around the implant structure. [6] There are many protocols for the effective management of peri-implantitis that include mechanical debridement, the use of antiseptics and local/systemic antibiotics, and access and regenerative surgery formulated for the treatment of peri-implantitis. Currently, there is no reliable evidence to recognize the most effective intervention method for treating peri-implantitis. [7] Surgical methods are widely employed to effectively manage moderate and advanced peri-implantitis. [8] One of the primary goals of surgical therapy is to get easy access to the implant surface decontamination. An anti-infective protocol that incorporates surgical access, surface decontamination, and systemic antimicrobials were proven effective in a 12 month follow-up. [9] Many reported regenerative procedures, using bone grafts or bone substitutes, sometimes combined with membranes, were targeted at reconstructing peri-implant osseous defects and have shown perplexing results. [10] It is predominantly accepted that the mild cases of peri-implantitis should be treated non-surgically; this approach is also recommended as the initial phase for severe cases. It usually consists of non-surgical debridement using specific implant curettes, sprayed glycine, and local antibiotics delivery if needed. [10] Various researchers suggest surgical treatment for cases in which limited improvement is evident after completing non-surgical treatment. Many surgical approaches have been proposed, including regenerative procedures, which are usually in combination with decontamination processes. [11] Therefore, it is of interest to evaluate the clinical outcomes of a mixed protocol for the regeneration of deep osseous defects.

## Materials and Methodology:

The study was conducted after obtaining approval from the ethical committee. Records obtained from 27 patients who had already received treatment for peri-implantitis on one or more implants were retrospectively examined with the proposed period between 24 and 30 months after their surgical treatment. A total of 33 implants were included and examined retrospectively. Patients were included as study participants if their records clearly showed that they had peri-implant bone defects on one or more implants which have been treated with the method detailed in this retrospective study. The records of those patients were considered excluded if there is any case of missing or incomplete pre-treatment, follow-up or final data, absent radiographs or surgical/regenerative protocols different from that described. Those patients who were treated non-surgically and those with Probing Depth (PD) of more than 6 mm and a lack of implant mobility were considered fit for surgical treatment. In order to be treated using regenerative surgical procedures, patients must be non-smokers or smoke cigarettes that are fewer than 10 per day.10 All the patients were advised to receive a professional hygiene session 2-3 days prior to the surgical treatment, and this was performed to decrease the intraoral bacterial load. The proposed treatment is comprised of a combination of surgical and antimicrobial therapy. All patients were allocated to receive antibiotic prophylaxis with 2 g amoxycillin 1 hour prior to the surgery. Before the procedure, all patients were advised to use 1 min mouth rinse with 0.2% chlorhexidine. Articaine 4% with 1:100,000 adrenaline was employed for the local anaesthesia by infiltration; a mandibular block was used in the mandible.

A sharp, clean incision was made on the crest and vertical releasing incisions on the buccal side of mesial and distal sides. Once the muco-periosteal flap was elevated, the granulation tissue around the affected implant was then removed with the help of surgical curettes. Once the implant and the associated osseous defects were exposed, a Teflon insert was mounted on a piezoelectric surgicaldevice used to remove the debris and calculus from the implant's surface. Following this, chemical decontamination was performed using a 35% phosphoric acid gel. The gel was left in place for about 2 minutes, then suctioned and thoroughly washed with copious irrigation with saline solution. The flap was then allowed to release by sharp incision on the buccal side to have a passive adaptation, and it was sutured using monofilament sutures. A combination of vertical mattress sutures and single interrupted sutures were then allowed for the flap stability and closure. Patients were informed to keep an ice pack at 10 minutes intervals and were advised not to brush on the treated area until 2 days after the removal of sutures. The post-op prescription mainly included: Amoxicillin + Clavulanic Acid 1 g tabs (twice a day) for 7 days, Metronidazole 250 mg tabs (thrice a day) for 7 days, Ibuprofen 600 mg tabs (twice a day) for 3 days, and chlorhexidine mouth rinse 0.2% (twice a day) for 15 days. Supplements like vitamin B complex and vitamin D were also being prescribed. Follow-up exams were then scheduled every 2 months for the first 6 months and then every 6 months unless required.

Descriptive statistics were calculated that include mean, SD, medians and confidence intervals at 95%. PD measurements at baseline level and one year at final follow-up were analysed using the one-way repeated measures ANOVA test, while the statistical analysis of the BoP and suppuration values at baseline and final evaluation was assessed using McNemar's test.

## Results:

All the baseline values have been briefed in the given table - 1. The study population consisted of 13 males and 14 females with a mean age of 53.8 when they received the surgical therapy. Nine patients were observed as smokers at the time of the surgical therapy, and 11 had a history of periodontitis. At the baseline, the mean Probing Depth was 8.19 ± 1.23 mm; Bleeding on Probing (BOP) was present on 29 out of 33 treated areas; pus was instead present on 9 out of 33 sites. At the time of surgical treatment, all patients were evaluated for ASA 1 and 2. They had received detailed written and oral information about the risks vs benefits of the treatment, and they had all given written informed consent. All patients had a confirmed diagnosis of peri-implantitis based on the presence of Bleeding on Probing (BOP) and/or pus, localized oedema, erythematosus peri-implant mucosa and radiographic signs of bone loss. Twenty-seven patients who had been surgically treated for peri-implantitis affecting 33 implants and followed for a minimum of 24 months were included in this retrospective evaluation; the mean follow-up time was 28.9 months (a range of 24-38 months). Post hoc tests using the Bonferroni correction revealed that the peri-implantitis treatment elicited a significant reduction in PD from the baseline to the one-year follow-up (8.19 ± 1.23 mm vs. 3.88 ± 0.724 mm, respectively), which was significant (p < 0.0005). At the time of final examinations, BOP was present on 9 out of 33 sites; pus was present only on two surgical sites. An exact Mc-Nemar’s test determined a statistically significant difference in the proportion of implants with BoP and pus pre and post intervention (p < 0.0005 and p = 0.001, respectively).

## Discussion:

The retrospective analysis in this study, 27 patients was managed with a mixed protocol based on the mechanical debridement, chemical/pharmacological decontamination and bone regeneration. The mean probing depth went from the initial 8.19 mm to 4.21 mm. One implant in the region of #18 had a negative outcome because the clinical conditions have considerably worsened with time and returned to the initial conditions. Moreover, all the other implants had noticeable improvement compared to the baseline. The PD reduction obtained as the results are already at 12 months and also at the final follow-up are satisfactory in terms of containment of the progression of the disease. Data relating to Bleeding on Probing and pus might confirm the reduction of inflammation and infection. Six patients developed some relapse of PD, but only in one of those with the probing depth observed severe (more than 8 mm), with the simultaneous presence of bleeding and pus was reported. In the remaining cases, the relapse was observed to be minimal (1 mm), with probing depth in all was ranged between 4 mm and 5 mm and the residual BOP is noticed in only one case.

The causes of failure could be estimated as many, from the patient’s non-compliance to the incomplete surface decontamination (where their complete sterility is neither predictable nor verifiable). [12] Moreover, the use of a grafting material which introduces an additional variable that can greatly determine a higher probability of failure if an infection occurs during healing. [13] The initial degranulation of the defects was conducted to completely expose the defect that resulted from bone resorption using a surgical curette first to perform a gross removal of the granulation tissue. A Teflon piezoelectric tip was then used to perform a meticulous removal on the implant surface without hampering it. The non-aggressiveness of the coated ultrasonic tips is elaborated both in vitro and in vivo. A study by Rühling et al. [14] displayed that the titanium surfaces treated with Teflon-coated ultrasonic tips were similar to the control surfaces and also that these inserts confirm reduced overheating. Recently, in a multi-centre study on 89 patients, Blasi et al. [15] elaborated that the plastic-coated tips provided statistically comparable results; after the ultrasonic degranulation of the defect, phosphoric acid was applied on the surface of the implants, making sure that the substance should not be in contact with hard and soft tissues. This was performed to further remove as much calculus residue as possible that usually remains between the micro-threads of the implants and to carry out the initial decontamination. In a very recent RCT by Hentenaar et al. [16] on 28 patients with implants affected by peri-implantitis, the application of 35% phosphoric acid was considered superior to mechanical debridement with rinsing using saline solution. Additionally, an in vitro study showed that implants which are treated with 37% phosphoric acid incubated with human blood mononuclear cells for 24 h inferred higher cell viability rates and an potent increase in the levels of IL-2, IL-4, IL-6, IL-10, and TNF-α, thus suggesting that it can modulate the immune response, thereby improving bio-functional processes and enhancing the success rate of dental implants. [17] In order to confirm the removal of any residual phosphoric acid, copious irrigation with saline solution was followed for 60 seconds, followed by a spray with 25µm glycine powder using a dedicated hand piece. Air polishing is also equally effective as implantoplasty and is less harmful on the surfaces than scaling with manual curettes or the sonic scaler. [18-19] Finally, to confirm the further disinfection of the implant surface and the bone defect surrounding it, tetracycline powder diluted in saline solution was applied to the entire defect. The dilution of tetracycline allows defect to avoid the potential decrease in cellular viability that this drug can be given at higher concentrations, thus favoring its antimicrobial and osteoblastic activities by inhibiting MMPs. [20] Tetracycline has often effectively proved to be an alternative aid in the decontamination of surfaces in peri-implant defects, in vitro and in vivo, precisely by its simultaneous antibiotic and inhibitory action of osteoclastic differentiation. [21] There is no reported evidence available in the literature to support the superiority of one treatment for peri-implantitis over another; [22] therefore, in our retrospective analysis of 27 cases and 33 implants, the authors have detailed how the combination of various strategies with the support of scientific evidence, lead to more than acceptable and maintainable results in the medium-term follow-ups. We could say that the combination of several chemical–mechanical decontamination methods had probably attributed to ensuring better results in maintaining clinical parameters within healthy ranges and might support the positive outcomes of regenerative therapy without any observable detrimental effects.

## Conclusion:

A combined chemical, mechanical and regenerative decontamination therapy is effective in the treatment of peri-implantitis. Further investigation, including a control group and/or histologic findings, might be needed to ascertain the clinical results reported in the clinical studies.
